# Synthesis and preclinical evaluation of [^11^C]uPSEM792 for PSAM^4^-GlyR based chemogenetics

**DOI:** 10.1038/s41598-024-51307-0

**Published:** 2024-01-22

**Authors:** Sridhar Goud Nerella, Sanjay Telu, Jeih-San Liow, Madeline D. Jenkins, Sami S. Zoghbi, Juan L. Gomez, Michael Michaelides, Mark A. G. Eldridge, Barry J. Richmond, Robert B. Innis, Victor W. Pike

**Affiliations:** 1 Molecular Imaging Branch, National Institute of Mental Health, National Institutes of Health, Bethesda, MD USA; 2 Biobehavioral Imaging and Molecular Neuropsychopharmacology Unit, National Institute on Drug Abuse, National Institutes of Health, Baltimore, MD USA; 3 Laboratory of Neuropsychology, National Institute of Mental Health, National Institutes of Health, Bethesda, MD USA

**Keywords:** Neural circuits, Nuclear chemistry

## Abstract

Chemogenetic tools are designed to control neuronal signaling. These tools have the potential to contribute to the understanding of neuropsychiatric disorders and to the development of new treatments. One such chemogenetic technology comprises modified Pharmacologically Selective Actuator Modules (PSAMs) paired with Pharmacologically Selective Effector Molecules (PSEMs). PSAMs are receptors with ligand-binding domains that have been modified to interact only with a specific small-molecule agonist, designated a PSEM. PSAM^4^ is a triple mutant PSAM derived from the α7 nicotinic receptor (α7^L131G,Q139L,Y217F^). Although having no constitutive activity as a ligand-gated ion channel, PSAM^4^ has been coupled to the serotonin 5-HT_3_ receptor (5-HT_3_R) and to the glycine receptor (GlyR). Treatment with the partner PSEM to activate PSAM^4^-5-HT_3_ or PSAM^4^-GlyR, causes neuronal activation or silencing, respectively. A suitably designed radioligand may enable selective visualization of the expression and location of PSAMs with positron emission tomography (PET). Here, we evaluated uPSEM792, an ultrapotent PSEM for PSAM^4^-GlyR, as a possible lead for PET radioligand development. We labeled uPSEM792 with the positron-emitter, carbon-11 (*t*_1/2_ = 20.4 min), in high radiochemical yield by treating a protected precursor with [^11^C]iodomethane followed by base deprotection. PET experiments with [^11^C]uPSEM792 in rodents and in a monkey transduced with PSAM^4^-GlyR showed low peak radioactivity uptake in brain. This low uptake was probably due to high polarity of the radioligand, as evidenced by physicochemical measurements, and to the vulnerability of the radioligand to efflux transport at the blood–brain barrier. These findings can inform the design of a more effective PSAM^4^ based PET radioligand, based on the uPSEM792 chemotype.

## Introduction

Chemogenetics aims to activate or inhibit specific neuronal populations in the brain by targeting an exogenously constructed receptor that is activated only by administration of a selective ligand^1–8^. This specificity makes chemogenetics a powerful tool for modulating neuronal signaling. Translation of this technology to humans has potential for the treatment of neuropsychiatric disorders. A means for assessing and monitoring chemogenetic receptor expression in animals or humans would greatly facilitate translation of chemogenetic technology into the clinic. Positron emission tomography (PET), in tandem with selective radioligands, is a powerful imaging technology that can provide full optimization and validation for successful translation^9^. Development campaigns based on clinically approved drugs can be a viable pathway to a useful PET radioligand.

The earliest chemogenetic systems were based on chemically engineered G protein-coupled receptors, the most popular being modified human muscarinic receptors^10^. These are called Designer Receptors Exclusively Activated by Designer Drugs (DREADDs) They are not activated by any endogenous ligand (*e.g*., not by acetylcholine for the muscarinic DREADD) but are responsive to certain exogenous agonists, such as clozapine-*N*-oxide (CNO), or deschloroclozapine^11–13^. However, these DREADD-activating ligands may also produce unwanted pharmacological responses that have effects on various cell-types. PET radioligands based on an exogenous activator may also have off-target binding in brain^14^. Improving the selectivity of DREADD activating ligands, or developing new chemogenetic systems with better ligand selectivity, will likely be beneficial for future clinical applications.

**Figure 1:**
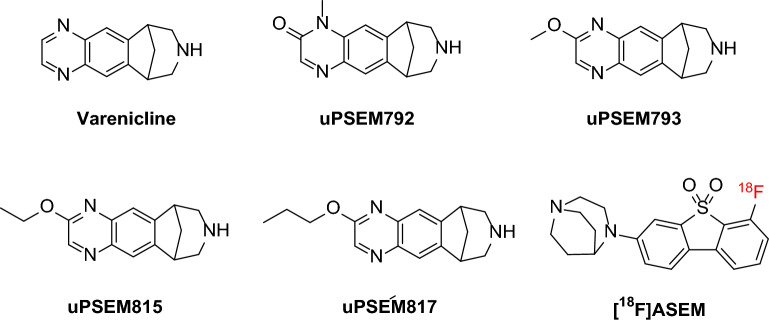
Structures of varenicline, uPSEM792, other uPSEMs, and [^18^F]ASEM.

Other chemogenetic modalities have been explored to overcome the limitations of DREADDs, especially their lack of selectivity. Structurally, PSAMs are chimeric ligand-gated ion channels (LGICs) comprising an α7 nicotinic acetylcholine receptor (α7-nAChR) ligand-binding domain and an ion pore domain of either the excitatory cation-selective 5-HT_3_ receptor (PSAM-5-HT_3_) or the inhibitory chloride-selective glycine receptor (PSAM-GlyR). A triple mutant PSAM (α7^L131G, Q139L,Y217F^), termed PSAM^4^ may be activated by a low dose of varenicline (Fig. 1), an α4β2-nAChR partial agonist and an FDA-approved smoking cessation drug^15^. PSAM^4^-GlyR reduces neuronal firing rate in the rhesus monkey in the globus pallidus internal region (GPi) when activated by varenicline^15^. Structural modification of varenicline has produced several ultrapotent and selective chemogenetic agonists (uPSEMs), such as uPSEM792, uPSEM793, uPSEM815, and uPSEM817 (Fig. 1)^15^. Neuronal silencing has been shown in area CA1 of mouse hippocampal neurons with uPSEM792 acting on PSAM^4^-GlyR^15^.

ASEM is an agonist at the endogenous α7-nAChR that has been labeled with a positron-emitter, fluorine-18 (*t*_1/2_ = 109.8 min) (Fig. 1)^16^. Moreover, [^3^H]ASEM has been used as the radioligand in binding assays for measuring the affinities of uPSEMs^15^. However, ASEM is not selective for PSAM^4^-GlyR because it has almost equal affinity for endogenous α7-nAChR (*K*_i_, 0.3 nM). PET imaging with [^18^F]ASEM has been used to visualize the localization of PSAM^4^-GlyR expression and binding of PSEMs to PSAM^4^-GlyR in mouse brain cortex^15^. However, [^18^F]ASEM was used at a very low molar activity (11 MBq/μmol)^15^, where molar activity (*A*_m_) is defined as the ratio of radioligand radioactivity to the total amount of ligand (radioactive plus non-radioactive) at a specified time. This low molar activity (high amount of carrier) may have avoided any interference from binding of the radioligand to endogenous nicotinic receptors or other off-target sites but may also have depressed target-specific signal^15^. Thus, a more selective and sensitive PET radioligand is desirable for verifying and imaging PSAM^4^-GlyR expression in animal models.

The affinity of a PET radioligand for its imaging target is a critical parameter in determining imaging performance^17^. Binding potential is proportional to the target density and to the binding affinity (reciprocal of *K*_D_ or surrogate value, such as *K*_i_). Typically, efficacious PET radioligands have affinities in the nanomolar to sub-nanomolar range. uPSEM792 has a high affinity (*K*_i_, 0.7 nM) for PSAM^4^-GlyR^18^. Concerning in vitro binding selectivity, uPSEM 792 is selective to PSAM^4^-GlyR over a panel of 48 endogenous receptors and transporters, has low affinity to α7-nAChRs, and almost tenfold lower affinity to α4β2-nAChR (*K*_i_, 5.3 nM) (Table 1). Moreover, uPSEM792 appeared not to be a P-gp substrate in vitro as judged by an efflux ratio of less than 2^15,18^. uPSEM792 was able to block [^18^F]ASEM binding in the brain of PSAM^4^-GlyR transduced mouse^15^.

**Table 1:** Physicochemical and pharmacological properties of selected ligands for PSAM^4^-GlyR and off-target endogenous α4β2-nAChR receptors.

Name	MW^a^ (Da)	tPSA^a^ (Å^2^)	Log*P*^a^	PSAM^4^-GlyR (*K*_i_, nM)^b^	α_7_-nAChR (*K*_i_, nM)^b^	α_4_β_2_ nAChR (*K*_i_, nM)^c^
Varenicline	211.11	36.8	0.91	1.3	–	0.11
uPSEM792	241.12	44.7	0.83	0.7	8000	5.3
ASEM	358.12	40.6	2.71	0.26	0.3	4000

^a^Values generated with ChemDraw.

^b,c^In vitro binding assay results are from the literature^15,16^.

Currently, the core structure of uPSEM792 is the only chemotype known to bind with ultrahigh affinity to PSAM^4^. Therefore this chemotype is the only available lead for PET radioligand development. We considered that [^11^C]uPSEM792 could be a candidate radioligand for imaging of PSAM^4^-GlyR in transduced brain in vivo. In this study, we sought to label uPSEM792 itself with carbon-11 (*t*_1/2_ = 20.4 min) for evaluation as a candidate PET radioligand for imaging of PSAM^4-^GlyR expression in animal models and to gain basic information on the properties of its chemotype for further PET radioligand development.

## Results

### Chemistry

**Scheme 1:**
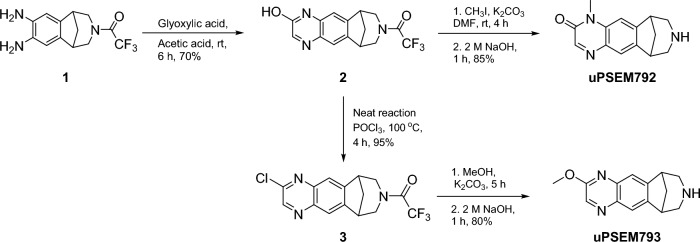
Synthesis of compound **2,** as a precursor for the ^11^C-labeling of uPSEM792, and reference compounds uPSEM792 and uPSEM793. Note that the products from **1** are racemic.

uPSEM792 and the *N*-protected hydroxy precursor **2** were synthesized in good yields from a commercially available aromatic diamine (**1**), according to reported methods^15^ with only minor modifications (see Supporting Information S1) (Scheme 1). To provide chromatographic reference material for the work-up of the radiosynthetic method, we synthesized uPSEM793 (Scheme 1) according to the reported method^15^, which is *O*-methylation of the chloro intermediate **3** followed by *N*-deprotection (see Supporting Information Sect.S1) (Scheme 1).

### Pharmacology

The affinity of uPSEM792 for PSAM^4^-GlyR was measured in a competitive binding assay using a [^3^H]ASEM as radioligand. uPSEM792 exhibited high binding affinity to PSAM^4-^GlyR (*K*_i_, 2.62 nM), similar to that found by Sternson et al. (*K*_i_, 0.7 nM)^15^. This result supported our decision to label uPSEM792 with carbon-11 and to evaluate [^11^C]uPSEM792 for imaging PSAM^4^-GlyR in vivo (Supporting Information, Fig. S4).

### Radiochemistry

**Figure 2:**
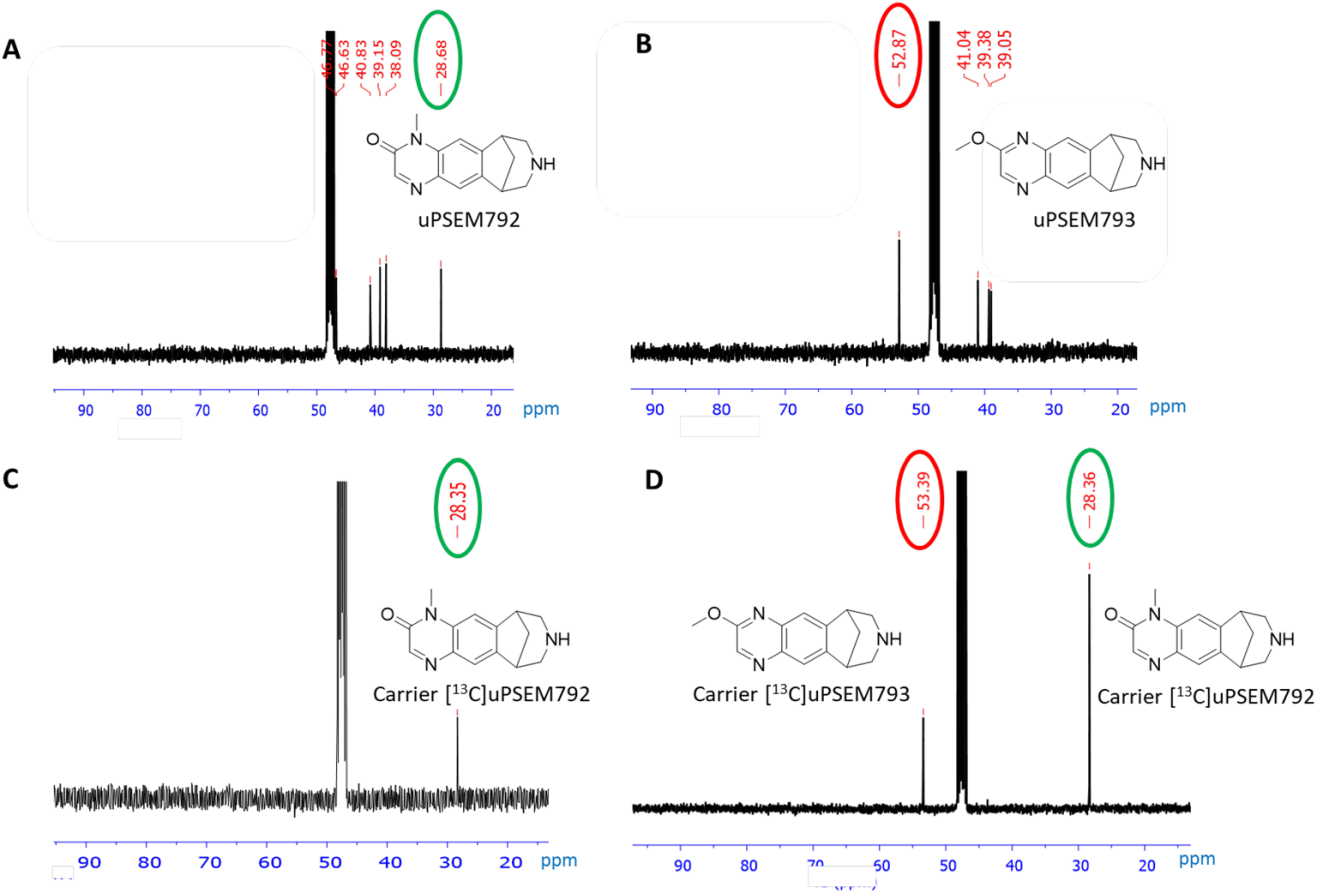
^13^C-NMR spectra (for aliphatic carbon region) verifying radiolabeling position in [^11^C]uPSEM792. (**A**) ^13^C-NMR spectrum of reference uPSEM792. (**B**) ^13^C-NMR spectrum of reference uPSEM793. (**C**) ^13^C-NMR spectrum of the carrier in [^11^C/^13^C]uPSEM792 isolated through HPLC from the reaction deploying [^11^C]iodomethane plus a known amount of [^13^C]iodomethane (conditions: K_2_CO_3_–K 2.2.2 at 80 °C in DMF). (**D**) ^13^C-NMR spectra of the carrier in [^11^C/^13^C]uPSEM792 and [^11^C/^13^C]uPSEM793 isolated through HPLC from the reaction deploying [^11^C]iodomethane plus a known amount of [^13^C]iodomethane (conditions: K_2_CO_3_–K 2.2.2 at 60 °C in MeOH).

**Scheme 2:**
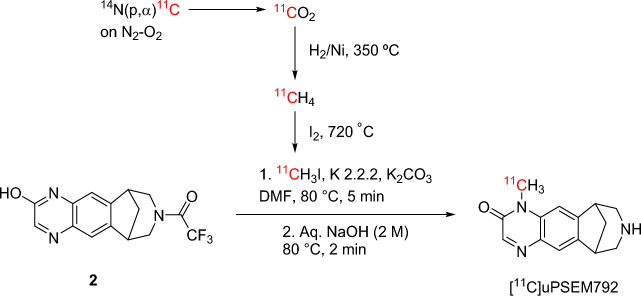
Radiosynthesis of [^11^C]uPSEM792.

We tested different bases, solvents, and reaction temperatures in the reaction of precursor **2** with [^11^C]iodomethane. To confirm that we produced [^11^C]uPSEM792 after base deprotection of the product from ^11^C-methylation, we conducted ^11^C-labeling experiments with a known amount of [^13^C]iodomethane as added carrier (in one tenth of the molar amount of precursor **2**), isolated the radioactive product peak from preparative HPLC, and analyzed the residual ^13^C-enriched carrier with ^13^C-NMR spectroscopy after almost full radioactive decay (i.e., after > 10 half-lives). The ^11^C-labeling site was verified by comparison of the ^13^C chemical shift of the main peak in the isolated carrier with the chemical shifts of *N*-methyl and *O*-methyl groups in the ^13^C-NMR spectra of uPSEM792 and uPSEM793 (namely, 28.7 and 52.9 ppm, respectively) (Fig. 2). In most trials, [^11^C]uPSEM792 was formed as the major radioactive product along with some [^11^C]uPSEM793. These two labeled compounds co-eluted under the initially employed HPLC separation conditions. However, they were separable under our analytical HPLC conditions. Finally, we found that treatment of compound **2** with [^11^C]iodomethane and K_2_CO_3_–K 2.2.2 as base in DMF at 80 °C for 5 min, followed by treatment with base (NaOH, 2 M) at the same temperature for 2 min (Scheme 2) produced [^11^C]uPSEM792 (*n* = 20) as a single radioactive product. [^11^C]uPSEM792 was readily isolated by reversed-phase HPLC (Supporting Information, Fig. S1). [^11^C]uPSEM792 was obtained from starting cyclotron-produced [^11^C]carbon dioxide (Scheme 2) in 45 min in useful overall yields (~ 14%, decay-corrected), high radiochemical purities (> 95%), and high molar activities (> 220 GBq/μmol). The identification of the isolated product as [^11^C]uPSEM792 was further supported by observation of its comobility with reference uPSEM792 on analytical HPLC (Supporting Information, Fig. S1). We used ascorbic acid in the formulation to prevent product radiolysis.

### In vitro stability and plasma free fraction

The stabilities of [^11^C]uPSEM792 for 30 min at 37 °C in homogenized non-perfused rat brain, whole blood, and plasma were measured. [^11^C]uPSEM792 appeared to be highly stable (94%) in rat brain. Stabilities were lower in whole blood (83.8%) and plasma (85.4%). The rat plasma free fraction (*f*_P_) was 89.8% and a pooled human standard plasma free fraction was 87.6%.

### Lipophilicity

The log*D*_7.4_ value of [^11^C]uPSEM792 was measured by partition between 1-octonol and sodium phosphate buffer (pH 7.4) and was found to be negative (− 1.40 ± 0.0; *n* = 6). We also measured log*D*_7.4_ with cyclohexane as an inert non-hydrogen bonding solvent in place of 1-octanol and obtained a value of − 3.85 ± 0.03 (*n* = 6). ΔLog*D*_*7.4*_, the difference between log*D*_7.4_ values obtained using octanol and cyclohexane (log*D*_7.4(octanol)_ − log*D*_7.4(cyclohexane)_) as the organic phase, was 2.45 ± 0.03 (*n* = 6).

### *pK*a of uPSEM792

The apparent *pK*_a_ of uPSEM792 was found to be 9.92 ± 0.05 (*n* = 3).

### PET imaging of uPSEM792 in rat

**Figure 3:**
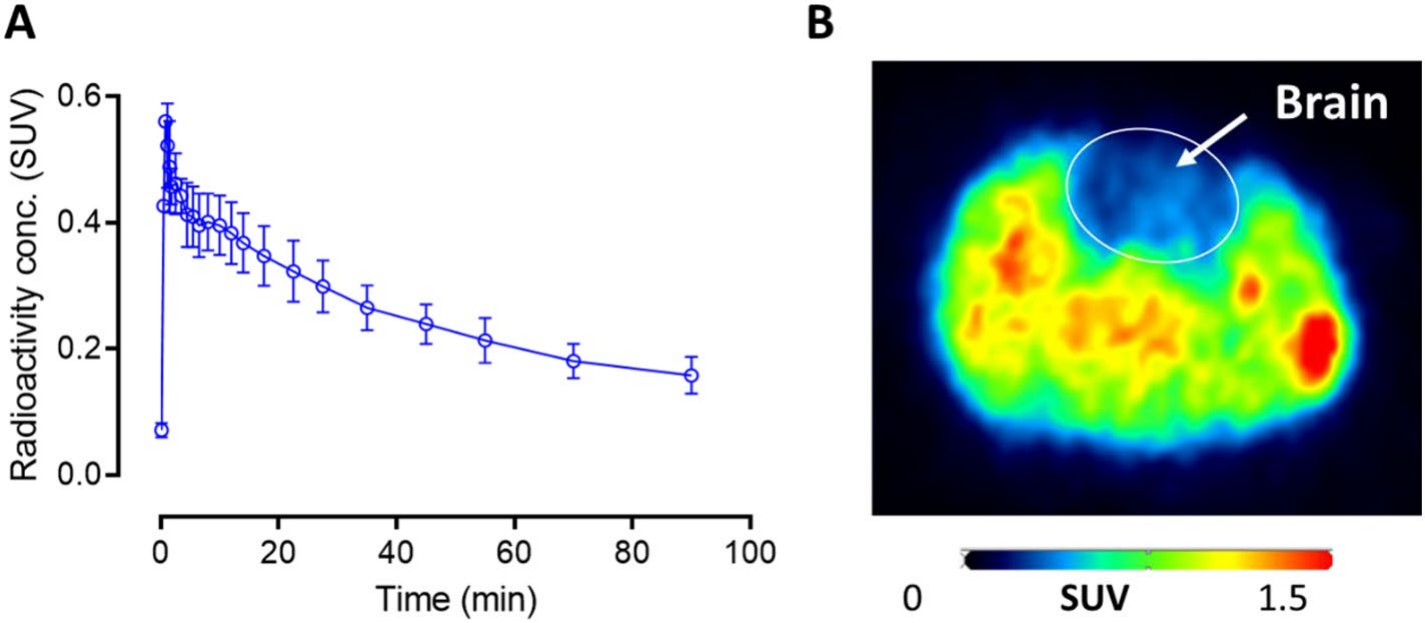
[^11^C]uPSEM792 has low uptake in wild-type rat brain. (**A**) Whole brain time-activity curve after intravenous injection of [^11^C]uPSEM792 into rats (mean ± range; *n* = 2). (**B**) Transaxial PET image of radioactivity concentration (SUV; summed from 0 to 120 min) of entire rat head including brain. Note the brain region, which is pointed out with the arrow, lies within the circle, and has conspicuously low radioactivity uptake.

PET imaging of brain was performed on wild-type rats after intravenous injection of [^11^C]uPSEM792. Radioactivity in whole brain peaked early (5 min) at a very low level (~ 0.6 SUV) and then rapidly declined (Fig. 3A).

### Ex vivo measurements of radioactivity after intravenous administration of [^11^C]uPSEM792 to rat

**Figure 4:**
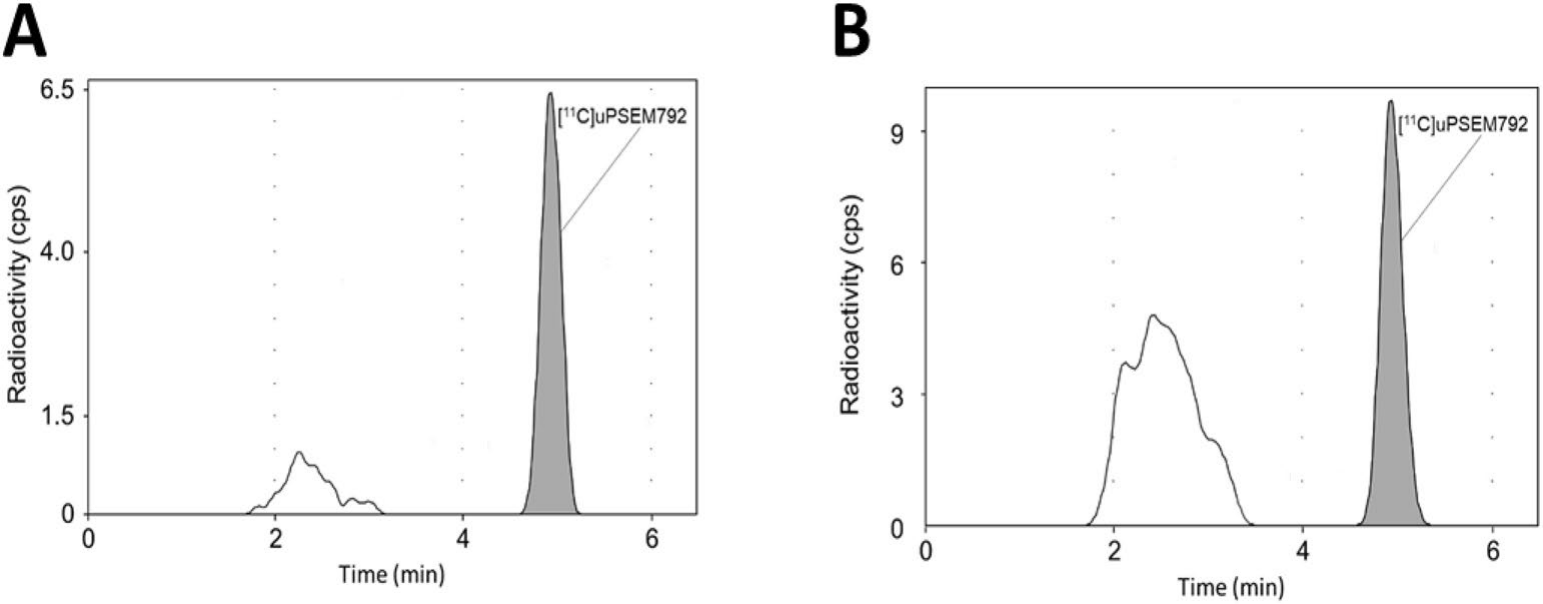
Intravenously administered [^11^C]uPSEM792 gives more polar radiometabolites in rat brain and plasma. Reversed phase HPLC radiochromatographic profiles are shown for rat brain (**A**) and plasma (**B**) at 100 min after intravenous administration of [^11^C]uPSEM792.

The total radioactivity concentration in rat brain at 100 min after radioligand injection was found to be low (SUV ~ 0.4) and consistent with the PET measurements. Unchanged radioligand represented 75.6% of the radioactivity in brain with the remainder composed of faster eluting (more hydrophilic) radiometabolites in the reversed phase HPLC analysis (Fig. 4A; Supporting Information, Table S1).

Unchanged radioligand fraction at 100 min was 37.9% of all radioactivity in plasma and was at a concentration of 0.064 ± 0.001 SUV (*n* = 3) corresponding to 6.7-fold lower than that in brain (Fig. 4B; Supporting Information, Table S1). The ratio of radioactivity concentration in blood cells to that in plasma was 2.4 ± 0.2 (*n* = 3).

Ex vivo confirmation of PSAM^4^-GlyR expression was not performed because the monkey is needed for longitudinal study. However, a previous study confirmed the expression of PSAM^4^-GlyR with [^18^F]ASEM and PET, and the use of uPSEM792 to show receptor specific binding^15^.

### PET imaging [^11^C]uPSEM792 in wild-type and efflux transporter knockout mice

**Figure 5:**
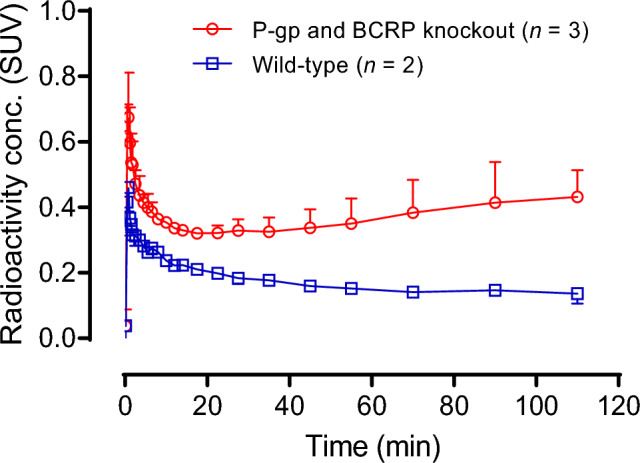
[^11^C]uPSEM792 gives higher average whole brain radioactivity uptake in dual efflux transporter knockout mice than in wild-type mice after *i.v.* injection of [^11^C]uPSEM792.

Early peak radioactivity uptake was about two-fold higher (~ 0.7 SUV) in dual efflux transporter (P-gp and BCRP) knockout mice than in wild-type mice, showing that [^11^C]uPSEM792 is a substrate for efflux transporters in vivo (Fig. 5). It has been reported that uPSEM792 is not a P-gp substrate in vitro^15^. However, the in vitro assay uses a high test concentration that may not be relevant to showing efflux transporter liability at very low radioligand concentrations in vivo. Moreover, uPSEM792 may have liability at other efflux transporters in the blood-brain barrier.

The time-activity curves in the knockout mice declined rapidly after reaching peak values before rising again after about 20 min. The time-activity curves in wild-type mice did not show this late rise.

### PET imaging of [^11^C]uPSEM792 in non-transduced healthy monkey

**Figure 6:**
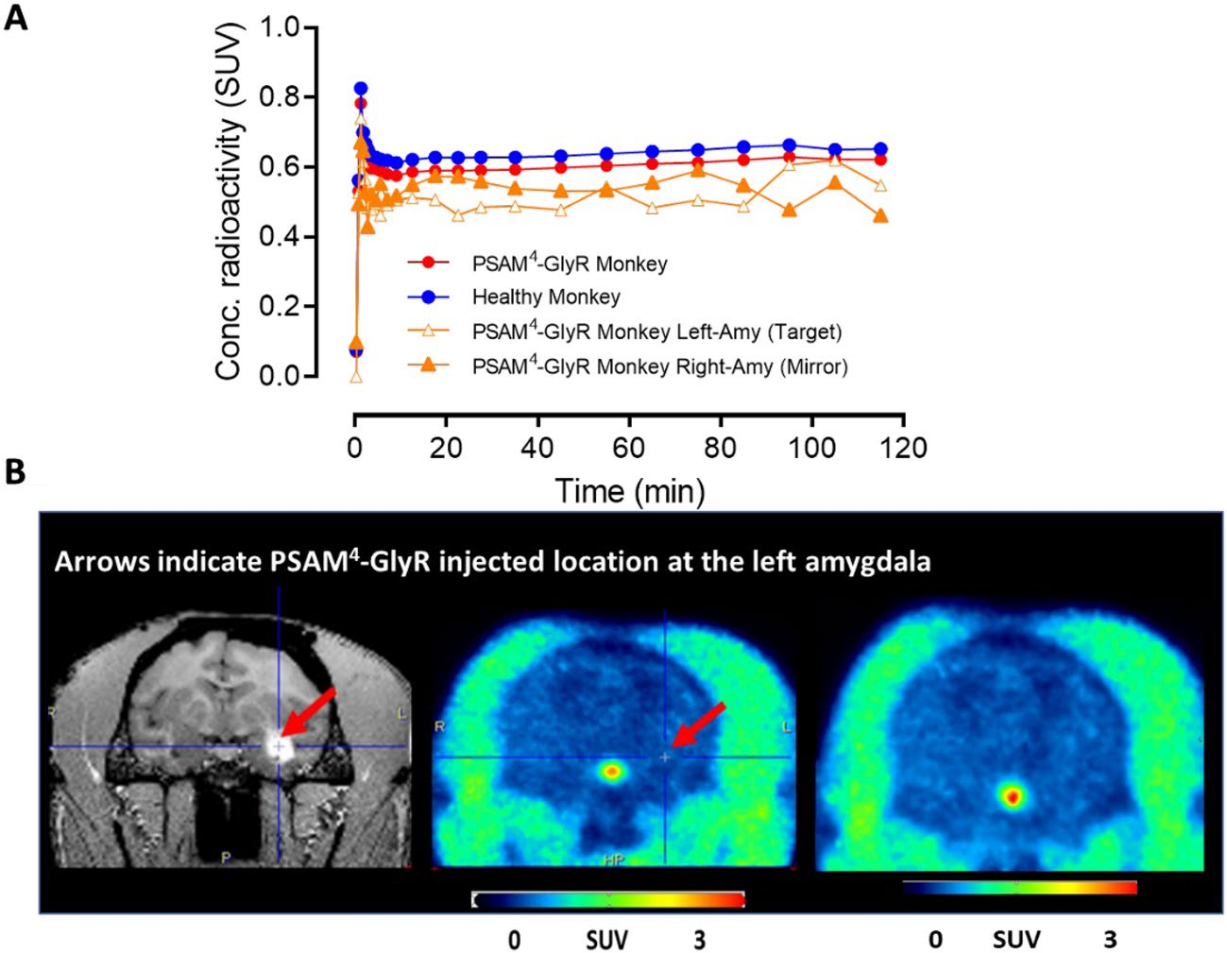
[^11^C]uPSEM792 has low uptake in non-transduced healthy and PSAM^4^-GlyR monkey brain. (**A**) Time-activity curves for whole brain after *i.v.* injection of [^11^C]uPSEM792 into non-transduced healthy and PSAM^4^-GlyR monkey, and for target (PSAM^4^-GlyR injected site, Left-amygdala), and mirror (opposite contralateral site, Right amygdala) (**B**) MRI (left) and PET images (summed from 0 to 120 min) for PSAM^4^-GlyR monkey whole brain (middle) and non-transduced healthy monkey whole brain (right).

The whole brain time-activity curve peaked at a low value (0.8 SUV), similar to that seen in rodent, and then slightly declined to a high plateau at about 0.65 SUV (blue solid circles, Fig. 6A and Supporting Information, Table S2).

### PET imaging in monkey expressing PSAM^4^-GlyR receptors

**Figure 7:**
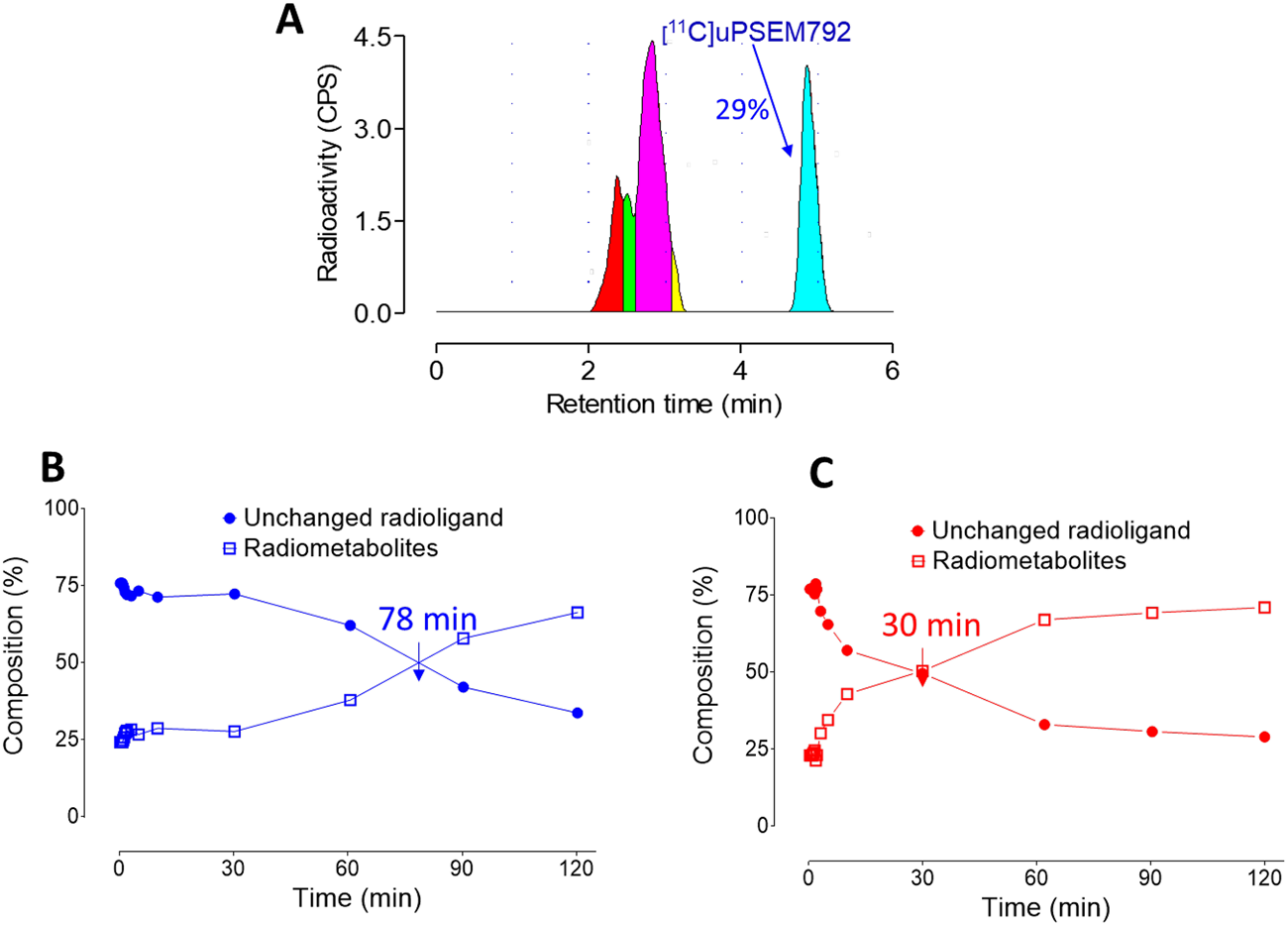
(**A**) Reversed phase HPLC analysis of non-transduced healthy monkey plasma sampled at 120 min after [^11^C]uPSEM792 injection showed that most of the radioactivity eluted as radiometabolites before unchanged radioligand. (**B**) By 78 min radiometabolites represented half of the radioactivity in plasma in non-transduced healthy monkey. (**C**) In PSAM^4^-GlyR monkey injected with [^11^C]uPSEM792, radiometabolites accounted for 50% of radioactivity in plasma at 30 min.

PET imaging with [^11^C]uPSEM792 in a monkey transduced in the left amygdala with PSAM^4^-GlyR showed a low peak whole brain radioactivity uptake (~ 0.8 SUV) followed by a slight decline to a plateau (red solid circles, Fig. 6A), as in non-transduced healthy monkey. No significant difference in the uptake of radioactivity between left and right amygdala was observed (orange solid triangles and orange empty triangles, Fig. 6A,B arrow mark and its mirror). The time-course for unchanged radioligand in plasma was similar to that in non-transduced healthy monkey and the concentrations (SUV) of unchanged radioligand. Radiometabolites were measured concurrently in arterial plasma to provide a radiometabolite-corrected arterial input function over the full-time course of PET scanning in both the naïve and PSAM^4^-GlyR transduced monkey (Supporting Information, Fig. S5). Radiochromatograms and radioactivity compositions for both non-transduced healthy and PSAM^4^-GlyR transduced monkey plasma are shown in Fig. 7. Radiometabolites were rapidly generated in the early phase of scanning but became an almost constant proportion of radioactivity in plasma after about 25 min. The low brain uptake values did not fit well with either one tissue or two tissue compartmental modeling. The data therefore do not reveal any evidence of specific binding in left amygdala.

## Discussion

uPSEM792 was considered to be an ultrapotent and selective PSAM^4^-GlyR candidate PET radioligand, based primarily on its high affinity (*K*_i,_ 2.62 nM) and amenability for labeling with carbon-11. [^11^C]Iodomethane is the most widely used synthon for labeling PET radiotracers with positron-emitting carbon-11^17^. We considered that the *N*-methyl group in uPSEM792 might be labeled by treating an *N*-desmethyl precursor with [^11^C]iodomethane. This approach requires protection of the secondary amino group. In fact, the original synthesis of uPSEM792 is through treatment of an *N*-trifluoroacetyl-protected hydroxy precursor (**2**) with iodomethane followed by mild base deprotection^15^, a method that we considered adaptable to ^11^C-chemistry.

Unlike the synthesis of uPSEM792 itself where precursor and iodomethane are equimolar, ^11^C-labeling typically requires the non-radioactive precursor to be present in several 100-fold excess over the ^11^C-labeling agent. Because the precursor **2** might undergo lactam-lactim (–NH–C=O ⇌ -N=C–OH) tautomerism^19^ under basic conditions, as would be used in radiolabeling, two radioactive tautomeric products might be formed, namely [^11^C]uPSEM792 and [^11^C]uPSEM793. Therefore, we performed comprehensive trials to find optimal conditions that would primarily produce the desired [^11^C]uPSEM792. We performed some carrier-added labeling experiments in which [^11^C]iodomethane was spiked with [^13^C]iodomethane to enable the labeling site in the radioactive product(s) to be determined by ^13^C-NMR spectroscopy (Fig. 2).

From these trials, we found that treatment of the precursor **2** with [^11^C]iodomethane and K_2_CO_3_–K 2.2.2 as base in DMF at 80 °C for 5 min followed by deprotection with a base (NaOH, 2 M) at the same temperature for 2 min (Scheme 2) produced [^11^C]uPSEM792 (*n* = 20) as a single radioactive product that was readily isolated by reversed phase HPLC. LC–MS/MS was also performed on [^11^C]uPSEM792 carrier, reference uPSEM792, and uPSEM793. This showed that carrier in [^11^C]uPSEM792 product has the same fragmentation pattern as reference uPSEM792 on MS/MS. This fragmentation pattern is distinct from that of uPSEM793 (Supporting Information, Figs. S2 and S3). This result corroborated the identity of the isolated [^11^C]uPSEM792. The establishment of an efficient radiosynthesis [^11^C]uPSEM792 in high radiochemical purity enabled us to proceed to physicochemical measurements, preclinical PET imaging, and ex vivo studies.

The findings of high rat plasma free fraction (89.8%) and pooled human standard plasma free fraction (87.6%) implied that [^11^C]uPSEM792 has very low lipophilicity^20^. In general, compound lipophilicity, as indexed by the distribution coefficient of the compound between 1-octanol and pH 7.4 sodium phosphate buffer (log*D*_7.4_), can be used to predict blood-brain barrier (BBB) permeability for drug-like molecules. For PET radioligands intended to image protein targets in brain, log*D*_7.4_ values between 1.0 and 3.5 are considered desirable^17^. However, the log*D*_7.4_ value of [^11^C]uPSEM792 was found to be negative (− 1.40 ± 0.0; *n* = 6), in accord with the measured high plasma free fractions^20^. The ability of 1-octanol to act as a hydrogen bond donor and acceptor influences the solubility of an uncharged solute species that has hydrogen bonding ability, as in the case of uPSEM792. Moreover, hydrogen bonding may decrease the passive diffusion of solutes across the blood-brain barrier^21^. Δlog*D*_7.4_, generally correlates inversely with passive blood-brain barrier penetration^22^. For [^11^C]uPSEM792, Δlog*D*_7.4_ is 2.45 ± 0.03 (*n* = 6). This large difference points to the strong hydrogen bonding ability of [^11^C]uPSEM792^22^.

uPSEM792 showed a high apparent *pK*_a_ (9.92 ± 0.05). Thus, only 0.02% of [^11^C]uPSEM792 would be uncharged at the physiological pH of plasma (7.4) and available to diffuse passively across the blood–brain barrier. The low brain uptakes of [^11^C]uPSEM792 in wild-type rats (< 0.6 SUV) and monkey (~ 0.8 SUV) are consistent with its negative log*D*_7._._4_, high Δlog*D*_7.4_, and high *pK*_a_ values. Effective radioligands for imaging protein targets in brain typically have peak uptakes well in excess of SUV 1^17^. Notwithstanding, we noted that the ratio of radioactivity concentration in blood cells to that in plasma was 2.4 ± 0.2 (*n* = 3) showing some ability of uPSEM792 to cross biological membranes.

P-glycoprotein (P-gp) and brain cancer resistance protein (BCRP) are the main efflux transporters for small organic-molecules at the blood–brain barrier, and they are known to restrict the brain uptake of several PET radioligands^17^. We imaged brains in wild type and dual P-gp and BCRP knockout mice after intravenous injection of [^11^C]uPSEM792 to assess its efflux transporter liability. The experiments showed that [^11^C]uPSEM792 is a substrate for efflux transporters, in contrast to findings from ex vivo assay^15,18^.

PET measures the time course of radioactivity distribution in vivo but gives no information on the nature of the composing radioactive molecular species. Ideally, the signal from brain for an intravenously administered PET radioligand should be derived from the unchanged radioligand only^23^. That is, signal should not be from any radiometabolites entering or formed in brain. Therefore, we carried out experiments in which radioactivity in brain and plasma was measured and analyzed at 100 min after intravenous injection of [^11^C]uPSEM792 into rat. Reversed-phase HPLC was used to separate unchanged [^11^C]uPSEM792 from radiometabolites. We found that a substantial percentage of the radioactivity in brain was composed of radiometabolites (24.4%), and also in plasma (62.1%). Radioligand metabolism tends to be faster in rodents that in higher species. The rodent finding was cautionary as to whether radiometabolites of [^11^C]uPSEM792 might be found in monkey brain^24^.

The brain uptake of PET radiotracers may also differ between species^17^. Therefore, we imaged non-transduced healthy monkey brain after intravenous injection of [^11^C]uPSEM792. We sampled arterial blood concurrently to analyze plasma for radiometabolites and enable the construction of a radiometabolite-corrected arterial input function. The whole brain time-activity curve peaked at 0.8 SUV, similar to that seen in rodent, and then slightly declined to a plateau (Fig. 6A). This plateau strongly suggested radiometabolite entry or entrapment within brain. High radioactivity uptake was seen in pituitary (Fig. 6B) as has been seen for some other PET radiotracers that have efflux transporter liability, such as [^11^C]*N*-desmethyl-loperamide^25^.

We considered that higher brain uptake of [^11^C]uPSEM792 might occur in the presence of transduced receptor targets. Therefore, PET imaging with [^11^C]uPSEM792 was also performed in a monkey with PSAM^4^-GlyR transduced into the left amygdala, MR imaging was used to locate the injected PSAM^4^-GlyR for PET radioactivity uptake analysis and the mirror region was used as the PSAM^4^-GlyR absent control. [^11^C]uPSEM792 showed a low peak whole brain radioactivity uptake (~ 0.8 SUV) (Fig. 6A), as in non-transduced healthy monkey. Radioactivity uptake in left and right amygdala showed no difference (Fig. 6B). The time-courses of radioactivity in arterial whole blood and of unchanged radioligand in arterial plasma were similar to those seen in non-transduced healthy monkey (Supporting Information, Fig. S5). The low brain uptake did not fit well with either a one tissue or two tissue compartmental model. Thus, no evidence was obtained for PSAM^4^-GlyR specific binding. A possible reason for lack of specific signal is that the local concentration of expressed PSAM^4^-GlyR (its *B*_max_ in nM units) is too low relative to the affinity of [^11^C]uPSEM792 (0.7 nM; Table 1). As a guide, this ratio normally needs to exceed a value of 5 for successful PET imaging of a brain protein.

## Conclusions

[^11^C]uPSEM792 was readily synthesized by ^11^C-methylation. Physicochemical measurements showed that [^11^C]uPSEM792 is quite polar and that this polarity with high H-binding capacity likely contributes to low brain entry in rodents and monkeys. Ex vivo measurements in rats after intravenous administration of [^11^C]uPSEM792 showed high concentration of unchanged radioligand in brain. Experiments in dual P-gp and BCRP knockout mice showed that [^11^C]uPSEM792 is a substrate for efflux transporters. Successful development of a PET radioligand for PSAM^4^-GlyR from the uPSEM792 platform will require extensive medicinal chemistry efforts to overcome these many limitations.

## Experimental section

### Materials and general methods

All purchased chemicals were used without further purification. uPSEM792 and uPSEM793 were synthesized as reference materials according to the literature^15^. The diamine precursor **2** was purchased from Toronto Research Chemicals Inc. (Toronto, Canada). Thin-layer chromatography was performed on silica gel plates (0.25 mm; 60 F_254_, Sigma Aldrich; Burlington, MA) and compounds were visualized with UV light (λ = 254 nm). Synthesized compounds were purified with flash column chromatography on silica gel. ^1^H-NMR spectra were obtained at 400 MHz and ^13^C-NMR spectra at 100 MHz on a Bruker (Billerica, MA) instrument, using *d*_*4*_-CH_3_OH, *d*_*6*_-DMSO or CDCl_3_ as solvent and tetramethylsilane as an internal standard. ^19^F-NMR were obtained at 376 MHz, using 4,4’-difluorobenzophenone as internal standard. Chemical shifts (*δ*) are reported in ppm and coupling constants are reported in Hz. The multiplicities of NMR signals are abbreviated as; s = singlet, d = doublet, t = triplet, m = multiplet, and dd = doublet of doublets*.* LC–MS spectra were acquired with an LCQ DECA instrument (Thermo Fisher Scientific; Waltham, MA) fitted with a Luna C18 column (55 μm; 2.0 × 150 mm; Phenomenex; Torrance, CA) eluted at 150 μL/min with MeOH-H_2_O (50:50 v/v). High resolution mass spectra (HRMS) were obtained with the ESI ionization method on Q-TOF-Mass analyzer operating at 70 eV using direct inlet. Melting points were recorded on an automated melting point apparatus (SMP30; 230 V AC; Stuart; TEquipment, NJ). Each compound was shown to have a chemical purity of > 95% by HPLC analysis.

Carbon-11 was measured using a calibrated ionization chamber (Atomlab 300; Biodex Medical Systems, Shirley, NY). All radioactivity measurements were corrected for physical decay. Radiochemical identity and purity were determined with reversed phase HPLC on a Sunfire C18 column (250 × 4.6 mm, 5 µm; Waters; Milford, MA) using a dual wavelength absorbance detector and a Flow-count radioactivity detector equipped with a NaI crystal (FC 3300 NaI PMT; BioScan; Wilmington, MA).

### Radiosynthesis of [^11^C]uPSEM792

All radiosyntheses were performed with an upgraded^26^ and remotely controlled Synthia-type apparatus^27^ in a lead-shielded hot-cell for protection of personnel from radiation. No-carrier-added [^11^C]carbon dioxide was produced by the ^14^N(p,α)^11^C nuclear reaction by irradiating nitrogen gas (164 psi) containing oxygen (1%) for 40 min with a proton beam (16.5 MeV, 45 μA) generated with a biomedical cyclotron (PETtrace 200; GE Healthcare; Milwaukee, WI). The [^11^C]carbon dioxide (~ 75 GBq) was then converted into [^11^C]iodomethane by reduction to [^11^C]methane with hydrogen over nickel at 350 °C followed by circulation through a heated (750 °C) iodine column^28^. Compound **2** (3.5 µmol, 1.0 equiv.) was treated with [^11^C]iodomethane in the presence of potassium carbonate (25 µmol, 7.0 equiv.) and kryptofix 2.2.2 (K 2.2.2.; 21 µmol, 6.0 equiv.) in DMF (400 µL) in a septum-sealed vial at 80 °C for 5 min. Aqueous sodium hydroxide (2 M, 200 µL) solution was then injected into the reaction mixture and heated at 80 °C for 2 min and then diluted with aqueous trifluoroacetic acid (0.1%; 1 mL). [^11^C]uPSEM792 was separated from the reaction mixture by single-pass reversed phase HPLC (Supporting information, Sect.  S4). The collected product fraction was reconstituted in 0.9% saline (10 mL) containing ethanol (9% v/v) and finally filtered through a sterile filter (Millex MP, 0.2 µm, Millipore Sigma; St. Louis, MO) for analysis and *i.v.* injection into monkey.

### Determination of radiochemical purity and molar radioactivity of [^11^C]uPSEM792

A sample (~ 12.5 MBq) of formulated [^11^C]uPSEM792 was analyzed with HPLC on a Sunfire C-18 column (5 µm; 4.6 × 250 mm; Waters, Milford, MA) eluted with MeCN-water (10:90 v/v) at 1.5 mL/min. Eluate was monitored for absorbance at 254 nm and for radioactivity. The mass of carrier uPSEM792 in the injectate was determined from a pre-calibrated mass response curve obtained under identical HPLC conditions. Molar activities (GBq/μmol) are reported as the radioactivity of [^11^C]uPSEM792 in the injected sample (GBq), divided by the mass of carrier uPSEM792 (μmol) at a specific time (Supporting Information; Fig. S1).

### In vitro radioligand binding assay

HEK-293 cells were transduced with plasmids (5 μg per dish) of encoding PSAM^4^-GlyR (in pcDNA3.1) or a control vector (GFP) and harvested 48 h after transduction. Cells were suspended in Tris–HCl buffer (50 mM, pH 7.4) supplemented with a protease inhibitor cocktail (1:100, Sigma-Aldrich; St Louis, MO). HEK-293 cells were disrupted with a Polytron homogenizer (Kinematica; Basel, Switzerland). Homogenates were centrifuged at 48,000*g* (50 min, 4 °C) and washed twice in the same conditions to isolate the membrane fraction. Protein was quantified by the bicinchoninic acid method (Pierce, ThermoFisher Scientific, MA). Membrane suspensions (50 μg of protein per mL) were incubated in Tris–HCl buffer (50 mM; pH 7.4) containing CaCl_2 (_8 mM), and [^3^H]ASEM (2 nM; 962,000 MBq/mmol, Novandi Chemistry AB; Södertälje, Sweden) and increasing concentrations of the tested compounds during 2 h at room temperature. Non-specific binding was determined in the presence of non-radioactive ASEM (1 μM). In all cases, free and membrane-bound radioligand were separated by rapid filtration of 500-μL aliquots in a 96-well plate harvester (Brandel; Gaithersburg, MD) and washed with ice-cold Tris–HCl buffer (2 mL). Microscint-20 scintillation liquid (65 μL per well, PerkinElmer; Boston, MA) was added to the filter plates. Plates were incubated overnight at room temperature and radioactivity counts were determined in a MicroBeta2 plate counter (PerkinElmer) with an efficiency of 41%. One-site competition curves were fitted using Prism 7 (GraphPad Software; La Jolla, CA). *K*_i_ values were calculated using the Cheng-Prusoff equation. The calculated *K*_d_ value of [^3^H]ASEM is 0.26 nM.

### Stereotaxic virus delivery

The procedure has been explained in the literature^29^.

### Transduction of PSAM^4^-GlyR in monkey brain

All studies were conducted in accordance with “Animal Research: Reporting of In Vivo Experiments” guidelines as well as Guidelines for the Care and Use of Laboratory Animals, 8th Edition, and were approved by the National Institute of Mental Health Animal Care and Use Committee. A lentivirus carrying an PSAM^4^ expressed under a human synapsin promoter (Lenti-hSyn-PSAM^4^-GlyR) was injected into the left amygdala of a single rhesus macaque (11-year-old male). 14 × 20 μL of virus was injected at 1.0 μL/min speed. The virus titer was measured at 1 × 10^9^ i.u./mL. The surgery was performed under aseptic conditions in a fully equipped operating suite.

### Magnetic resonance imaging

A magnetic resonance imaging (MRI) scan was performed after brain injection using a 3 T MRI scanner (Achieva dStream, Philips Healthcare, Best, Netherlands), as previously described^30^. For all MRI procedures, anesthesia was performed with ketamine (10 mg/kg, i.m.) and dexmedetomidine (0.2 mL, i.m.).

### In vitro stability and plasma free fraction estimation

In vitro stability was calculated by dividing the relative composition of the blood or brain homogenate determined with radio-HPLC by the initial radiochemical purity of the radioligand. The plasma free fraction (*f*p) of [^11^C]uPSEM792 in fresh rat plasma and in human pooled standard plasma was measured by ultrafiltration through membrane filters (Centrifree; Millipore, St. Louis, MO), as previously described^24,31^. Briefly, formulated [^11^C]uPSEM792 (1.85 MBq; ~ 5 µL) was added to plasma (700 µL). The mixture was incubated at room temperature for 10 min and then processed exactly as described previously. The ultrafiltration components that contained high radioactivity were allowed time for radioactivity decay to within the optimal range of the γ-counter before they were counted again.

### Lipophilicity measurement

[^11^C]uPSEM792 (~ 266 MBq) in ethanol (550 µL) was added to phosphate buffer (0.15 M; 7 mL) and mixed well. Aliquots were distributed to 16 borosilicate disposable culture tubes (13 × 100 mm). The tubes were divided into two groups of 8. Buffered [^11^C]uPSEM792 solutions from 6 tubes were extracted with 1-octanol and another six were extracted with cyclohexane. Extraction was performed by vortexing the tubes with their content for 1 min. The tubes were then centrifuged for 1 min. The organic and the aqueous phases were separated, and each phase was then sampled (50 µL) and counted separately in a well-type γ-counter (model 1480 Wizard; Perkin-Elmer) with an electronic window set between 360 and 1800 keV. The remaining two tubes, containing the radioactivity in buffer, served as stability measures for the radioligand. Radioactivity in the aqueous phases (50 µL) resulted in a counting error of 1.2 ± 0.05% (*n* = 6) in the 1-octanol group and 0.4 ± 0.01% (*n* = 6) in the cyclohexane group, at one standard deviation. The aqueous phases were injected onto the HPLC. The resulting radiochromatograms provided correction factors for the γ-counter counts, of the aqueous samples, to determine parent radioligand radioactivity only (corrected radioactivity)^32^. Log*D*_7.4octanol_ and log*D*_7.4cyclohexane_ were each calculated as log((cpm in organic phase)/(corrected cpm in aqueous phase)).

### Measurement of apparent p*K*_a_

The method was similar to that followed previously for other radiotracers^33^. Sodium phosphate buffers (0.15 M), with pH values of 3.0, 3.5, 4.0, 4.5, 5.0, 5.5, 6.0, 6.5, 7.0, 7.4, 8.0, 8.5, 9.0, 9.5, 10.0, and 10.5, were prepared from 0.15 M NaH_2_PO_4_ and 0.1 M Na_2_HPO_4_ and distributed (1 mL per tube) as the aqueous phase. [^11^C]uPSEM, formulated in ethanol (20 µL, 2.4 ± 0.3 MBq, *n* = 48), was added to each tube. Triple sets of such tubes were prepared simultaneously. Cyclohexane (1.0 mL) was added to each of the tubes containing the buffer and the [^11^C]uPSEM and then vigorously vortexed for 1 min. The tubes were then centrifuged for one minute. The two phases in each tube were separated, sampled (50 µL aqueous, 200 µL organic) and counted in a γ-counter. Distribution coefficients at each pH were calculated by dividing the counts detected in the organic phase by the total activity present in both phases. These distribution coefficients were plotted versus pH and nonlinear regression analysis applied with GraphPad Prism version 4.03 for windows (GraphPad Software; San Diego, CA) using “One site competition” curve-fitting. The apparent p*K*_a_ was taken to be the pH value where the concentration of the ionized and non-ionized species of [^11^C]uPSEM are equal. The radiochemical purity of the [^11^C]uPSEM preparation was 98.8% as determined by radio-HPLC on an X-Terra C11 column (10 µm, 7.8 × 300 mm; Waters Corp.) and a mobile phase of MeOH:H_2_O:Et_3_N (55:45:0.1, by vol) at 5.0 mL/min.

### Ex vivo experiments

An ethanol solution of [^11^C]uPSEM792 (~ 42.55 MBq) in 0.9% saline (10% v/v) was injected intravenously through the penile vein of each of three rats that had been anesthetized with 1.5% isoflurane (Supporting Information, Table S1). Other experimental parameters that governed this study are listed in Supporting Information, Table S1. One of the rats was treated with blocking agent (uPSEM792, 1.5 mg/kg *i.v.*, formulated with 10% EtOH, and 90% saline) at 15 min before radioligand injection. The rats were sacrificed via thoracotomy 100 min after radioligand injection. Anticoagulated blood was drawn from the myocardium followed by decapitation and harvesting of the brain. Each brain was removed, weighed, and immediately subjected to radio analysis after counting its concomitant radioactivity. Whole blood samples were removed and centrifuged to separate the plasma. Whole blood (300 µL each) and plasma samples (300 µL each) were also quantified for radioactivity in an automatic γ-counter (model 2480 Wizard 2; Perkin-Elmer) with an electronic window set at 360–1800 keV (counting efficiency, 51.84%). Briefly, plasma samples (~ 450 µL) were deproteinated with acetonitrile treatment (720 µL). The radioactivity of various plasma and brain tissues were counted in the γ-counter. Brain tissues were then homogenized in acetonitrile (1.0 mL) using a handheld tissue Tearor (model 985-370; BioSpec Products Inc; Bartlesville, OK), followed by a second homogenization in water (1.0 mL). The homogenates were counted in the γ-counter to calculate the percentage recovery of radioactivity in the acetonitrile extracts. Corrections of all tissue assays for physical decay were made with a half-life of 20.385 min. The homogenates were then centrifuged at 10,000*g* for 2 min. The clear supernatants were injected onto the HPLC column through nylon filters. For each sample, radioactivity in the resulting precipitate was used to calculate the percent recovery of activity into the acetonitrile supernatant.

Five milliliters of anticoagulated blood (STD-A) and brain (1.8 g) were harvested from a wild-type rat (~ 450 g) and used in the in vitro assays. The brains were immediately weighed and homogenized with twice their weights (3.6 mL) of ice cold 0.9% NaCl solution. Plasmas were separated from whole blood via centrifugation. A radioligand amount (1.66 MBq in 5 µL) was then added to these three tissue samples before incubation in a shaking water bath at 37 °C for 60 min. Following incubation, aliquots (450 µL) were removed for radio-analysis and processed as detailed above for radio-HPLC analysis.

Anticoagulated blood (STD-A; 5 mL) and brain (1.8 g) were harvested from a wild-type rat (~ 450 g) and used for the in vitro assays. The brains were immediately weighed and homogenized with twice their weights (3.6 mL) of ice-cold 0.9% saline. Plasma was separated from whole blood via centrifugation. A radioligand amount (1.66 MBq in 5 µL) was then added to these three tissue samples before incubation in a shaking water bath at 37 °C for 60 min. Aliquots (450 µL) were then removed for radio analysis and processed as detailed above for radio-HPLC analysis.

### PET imaging

All PET imaging studies were performed with a Mediso LFER 150 preclinical scanner (nanoScan PET/CT). Brain and brain region radioactivity uptake was expressed by standardized uptake value (SUV) which normalizes for injected radioactivity and monkey body weight, as follows: SUV = [(% injected dose per g tissue) × body weight in g]/100.

#### Rat imaging

Three rats (body weight 376 ± 5 g) were scanned in a single session with [^11^C]uPSEM792 (injected activity 43.47 ± 42 MBq). Two were wild-type and one was pre-blocked with uPSEM792 (2 mg/kg, administered intravenously 15 min before radioligand). Animals were maintained under anesthesia with 1.5% isoflurane throughout the scan. Radioligand was injected intravenously via a penile vein catheter followed by PET imaging for 90 min. Brain uptake was expressed as standardized uptake value (SUV, concentration normalized by injected activity and body weight) and compared between the wild-type and self-blocked conditions.

#### Mouse imaging

To check for possible transporter substrate behavior for [^11^C]uPSEM792 at the blood-brain barrier, three wild-type and three double transporter knockout (P-gp and BCRP) mice (body weight 40.2 ± 7 g) were imaged in a single PET session (injected activity 7.28 ± 40 MBq). Radioligand was injected intravenously via a tail vein catheter followed by PET imaging for 90 min. Whole brain uptake in SUV was compared between wild type and knockout mice.

#### Monkey imaging

Two male rhesus monkeys (*macaca mulatta*; 10.0 and 8.6 kg) underwent PET scanning. Each monkey was initially immobilized with ketamine (10 mg/kg, i.m.) and then maintained under anesthesia with 1.5% isoflurane. For each monkey, a baseline scan was performed for 120 min. Upon each intravenous injection of [^11^C]uPSEM792 (0.18–0.24 GBq) at molar activities in the range 38–165 GBq/µmol, serial arterial blood sampling via an indwelling port was commenced to obtain a radiometabolite-corrected input function for PET data quantification. Electrocardiogram, body temperature, heart, and respiration rates were monitored throughout the scans.

Total radioactivity concentrations in sampled arterial whole blood and plasma were measured in a γ-counter throughout the 90-min scanning period. The percentages of radioactivity in plasma samples represented by unchanged [^11^C]uPSEM792 were also measured with radio-HPLC on a reversed phase HPLC column (XTerra, 10 µm, 7.8 × 300 mm; Waters Corp.) eluted at 4.5 mL/min with MeOH:H_2_O:Et_3_N (80:20:0.1 by vol.).

### Image processing

PET images were reconstructed using Fourier rebinding plus two-dimensional filtered back-projection with attenuation and scatter correction. Images were co-registered to a standardized monkey MRI template using the FMRIB Software Library (FSL; Oxford, UK). Thirty-four predefined brain regions of interest from the template were applied to the co-registered PET image to obtain regional, decay-corrected, time-activity curves.

### Animal care

All PET imaging experiments in rodents and monkeys were performed in accordance with the Guideline for Care and Use of Laboratory Animals^34^ and were approved by the National Institute of Mental Health Animal Care and Use Committee.

### Supplementary Information

Supplementary Information.

## Data Availability

The datasets generated during and/or analyzed during the current study, other than those reported in the Supporting Information, are available from the corresponding author on reasonable request.
