# Supplementary material for: Synthesis and preclinical evaluation of [11C]uPSEM792 for PSAM4-GlyR based chemogenetics
Source: Sci Rep. 2024 Jan 22;14:1886. doi: 10.1038/s41598-024-51307-0 (PMC10803328; doi:10.1038/s41598-024-51307-0)
Supplement: Supplementary file 1 — Supplementary Information. [file 41598_2024_51307_MOESM1_ESM.docx]

Supporting Information

**[Synthesis and Preclinical Evaluation of [](https://www.science.org/doi/10.1126/science.aav5282)^[11](https://www.science.org/doi/10.1126/science.aav5282)^[C]uPSEM792 for PSAM](https://www.science.org/doi/10.1126/science.aav5282)^[4](https://www.science.org/doi/10.1126/science.aav5282)^[-GlyR Based Chemogenetics](https://www.science.org/doi/10.1126/science.aav5282)**

Sridhar Goud Nerella^a^, Sanjay Telu^a*^, Jeih-San Liow^a^, Madeline D. Jenkins^a^, Sami S Zoghbi^a^, Juan L. Gomez ^b^, Michael Michaelides^b^, Mark G. Eldridge^c^, Barry J. Richmond^c^, Robert B Innis^a^, and Victor W Pike^a*^

^a^ Molecular Imaging Branch, National Institute of Mental Health, National Institutes of Health, Bethesda, MD, USA.

^b^ Biobehavioral Imaging and Molecular Neuropsychopharmacology Unit, National Institute on Drug Abuse, National Institutes of Health, Baltimore, MD, USA.

^c^ Laboratory of Neuropsychology, National Institute of Mental Health, National Institutes of Health, Bethesda, MD, USA.

**Contents**

1. Chemistry………………………………………………………….………………… 3

2. NMR spectra……………………………………………………….………………… 6

3. HPLC purity………………………………………………………………………… 11

4. Purification and formulation of radioligand ………………..………………....…… 11

5. HPLC radiochromatograms of radioligand…………………...…………………… 12

6. LC-MS/MS-MS spectra of carrier in [^11^C]uPSEM792.………………………. 14

7. In-vitro binding affinity studies……………………………………………………… 16

8. *Ex vivo* studies*….*…………………………………………..…………...…… 16

9. PET imaging in monkeys: experimental parameters………………………………… 17

10. PET imaging studies……………………………………….…………………..…… 18

**1. Chemistry**

**2,2,2-Trifluoro-1-2-hydroxy-6,7,9,10-tetrahydro-8*H*-6,10-methanoazepino[4,5-g]quinoxalin-8-yl)ethan-1-one (2).**

To a solution of glyoxylic acid (6.5 mmol, 1.2 equiv.) in DMF (15 mL) was added acetic acid (10.0 mmol, 2.0 equiv.) followed by 1-(7,8-diamino-4,5-dihydro-1*H*-1,5-methanobenzo[d]azepin-3(2H)-yl)-2,2,2-trifluoroethanone (1) (5.0 mmol, 1.0 equiv.) at room temperature. The mixture was then stirred for 5 h with reaction monitored by TLC. Ice-cold water was then added (50 mL) and stirred for 30 min, and then filtered, and dried under *vacuo* for 45 min. The obtained residue was purified by flash chromatography using mobile phase A of ethyl acetate and mobile phase B of hexane to afford compound **2** as an off white solid; yield 70%; Mp 212–214 °C; ^1^H NMR (400 MHz, DMSO) *δ* 12.47 (s, 1H), 8.10 (s, 1H), 7.66 (d, *J* = 8.2 Hz, 1H), 7.22 (d, *J* = 15.4 Hz, 1H), 4.16 (d, *J* = 12.3 Hz, 1H), 3.78 (s, 1H), 3.69 (d, *J* = 12.6 Hz, 1H), 3.43 (d, *J* = 5.9 Hz, 2H), 3.26 (d, *J* = 12.3 Hz, 1H), 2.24 (d, *J* = 4.9 Hz, 1H), 2.09 (d, *J* = 10.9 Hz, 1H). ^13^C NMR (100 MHz, DMSO) δ 156.52, 156.18, 155.44, 155.42, 150.72, 148.45, 148.01, 140.35, 139.79, 131.97, 131.89, 123.12, 122.99, 117.97, 115.10, 110.37, 110.27, 50.39, 48.22, 48.07, 41.64, 39.21, 39.04. ^19^F NMR (376 MHz, *d*_6_-DMSO) *δ* –67.63 ppm; HRMS (*m*/*z*): calculated for C_15_H_12_F_3_N_3_O_2_, 323.0960, found 324.0955 [*M*+H]^+^.

**1-(2-Chloro-6,7,9,10-tetrahydro-8*H*-6,10-methanoazepino[4,5-*g*]quinoxalin-8-yl)-2,2,2-trifluoroethan-1-one (3):**

Phosphorus oxychloride (3.5 mL, 6 equiv.) was added to compound **2** (2 g, 1 equiv.) and then the reaction mixture was stirred under reflux at 100 ^o^C for 3 h, cooled to room temperature, slowly poured into ice water, and then extracted thrice with dichloromethane. The combined organic layers were washed with saturated aqueous NaHCO_3_ solution and then brine solution, and then dried over Mg_2_SO_4_, and concentrated under *vacuo* to give **3** as a cream solid (1.9 gm, 95% yield); Mp 241–243 °C; ^1^H NMR (400 MHz, CDCl_3_) δ 8.76 (d, *J* = 5.5 Hz, 1H), 7.94 (s, 1H), 7.85 (s, 1H), 4.51 (d, *J* = 12.8 Hz, 1H), 4.08 (d, *J* = 12.5 Hz, 1H), 3.70 (d, *J* = 12.6 Hz, 1H), 3.57 (d, *J* = 19.2 Hz, 2H), 3.30 (d, *J* = 13.0 Hz, 1H), 2.61 – 2.46 (m, 1H), 2.18 (d, *J* = 11.3 Hz, 1H). ^13^C NMR (101 MHz, CDCl_3_) δ 157.05, 148.37, 147.81, 147.21, 146.99, 146.68, 144.21, 142.30, 141.23, 122.78, 122.05, 117.49, 114.63, 50.76, 48.52, 41.82, 39.99, 39.75. ^19^F NMR (376 MHz, CDCl_3_) *δ* –68.5 ppm; HRMS (*m*/*z*): calculated for C_15_H_11_ClF_3_N_3_O, 341.0621, found 342.0616 [M+H]^+^.

**1-Methyl-1,6,7,8,9,10-hexahydro-2*H*-6,10-methanoazepino[4,5-g]quinoxalin-2-one (uPSEM792):**

To a solution of 2,2,2-trifluoro-1-(2-hydroxy-6,7,9,10-tetrahydro-8*H*-6,10-methanoazepino[4,5-g]quinoxalin-8-yl)ethan-1-one (**2**) (0.25mmol, 1 equiv.), in DMF (2 mL) was added iodomethane (0.5 mmol, 2 equiv.) and potassium carbonate (1.0 mmol, 4 equiv.). The reaction mixture was then stirred at room temperature for 4 h. After confirmation of reaction by TLC, aqueous sodium thiosulfate (1 M; 50 mL) solution was added, and extracted with thrice with ethyl acetate. The combined organic layers were concentrated under *vacuo*. The resultant crude oil was mixed with sodium hydroxide solution (2 M, 0.5 mL) and methanol (3 mL), and then heated at 80 °C for 30 min. Then volatile components of the reaction mixture were removed under *vacuo*. The residue was purified b*y* flash chromatography using mobile phase A (dichloromethane) M and mobile phase B comprising a mixture of methanol, triethylamine, and DCM (10: 1: 89 by vol.) to afford uPSEM792 as a brown solid; yield 85%; Mp 225–227 °C; ^1^H NMR (400 MHz, MeOD, D_2_O) δ 8.24 (s, 1H), 7.87 (s, 1H), 7.70 (s, 1H), 3.77 (s, 3H), 3.59 (d, *J* = 12.4 Hz, 2H), 3.48 (ddd, *J* = 12.3, 6.8, 1.5 Hz, 2H), 3.30–3.23 (m, 2H), 2.52–2.42 (m, 1H), 2.22 (d, *J* = 11.5 Hz, 1H). ^13^C NMR (101 MHz, MeOD) δ 155.62, 148.63, 146.65, 138.35, 133.84, 133.61, 124.66, 110.46, 46.77, 46.63, 40.83, 39.15, 38.09, 28.68; HRMS (*m*/*z*): calculated for C_14_H_15_N_3_O, 241.1293, found 242.1289 [M+H]^+^.

**2-Methoxy-7,8,9,10-tetrahydro-6*H*-6,10-methanoazepino[4,5-*g*] quinoxaline (uPSEM793):**

Potassium carbonate (1.0 mmol) was added to a solution of **3** (0.25mmol in methanol (4 mL) and then stirred at 80 °C overnight. After confirmation of reaction by TLC, aq. sodium hydroxide solution (2 M, 0.5 mL) was added and heated to 80 °C for 30 min. Volatile components of the reaction were then removed under *vacuo*. The residue was purified by flash chromatography using mobile phase A (dichloroetnane) and mobile phase B (methanol,(triethylamine, DCM; 10, 1, and 89 by vol.) to afford uPSEM793 as a light yellow solid; yield 80%; Mp 205–207 °C; ^1^H NMR (400 MHz, MeOD) δ 8.45 (s, 1H), 7.93 (s, 1H), 7.83 (s, 1H), 4.10 (s, 3H), 3.53 (s, 2H), 3.41 (d, *J* = 12.0 Hz, 2H), 3.22 (d, *J* = 12.2 Hz, 2H), 2.47 (d, *J* = 5.1 Hz, 1H), 2.22 (d, *J* = 11.4 Hz, 1H). ^13^C NMR (101 MHz, MeOD) δ 157.96, 146.03, 142.15, 141.13, 138.79, 138.37, 122.71, 121.62, 52.87, 41.04, 39.38, 39.05; HRMS (*m*/*z*): calculated for C_14_H_15_N_3_O, 241.1293, found 242.1289 [*M*+H]^+^.

**2. NMR Spectra**

^1^H NMR spectrum of compound **2** at 400 MHz (d_6_-DMSO).

^13^C-NMR spectrum of compound **2** at 101 MHz (d_6_-DMSO).

^19^F NMR spectrum of compound **2** at 376 MHz (d_6_-DMSO).

^1^H NMR spectrum of compound **3** at 400 MHz (CDCl_3_).

^13^C NMR spectrum of compound **3** at 101 MHz (CDCl_3_).

^19^F NMR spectrum of compound **3** at 376 MHz (CDCl_3_).

^1^H NMR spectrum of uPSEM792 at 400 MHz (d_4_-MeOH & D_2_O).

^13^C-NMR spectrum of uPSEM792 at 101 MHz (d_4_-MeOH).

^1^H NMR spectrum of uPSEM793 at 400 MHz (*d*_4_-MeOH & D_2_O).

^13^C-NMR spectrum of uPSEM793 at 101 MHz (*d*_4_-MeOH).

**3. HPLC Purity**

HPLC chromatogram of uPSEM792 (*t*_R_, 9.8 min**).** Conditions; Sunfire C18 column (5 µm; 150 mm × 4.6 mm) eluted with 0.1% trifluoroacetic acid-MeCN (90:10 v/v) at 1 mL/min.

**4. Purification and Formulation of Radioligand.**

4.1. HPLC purification conditions for [^11^C]uPSEM792.

Sunfire 5 µm C18-OBD (250 mm × 10 mm) eluted (*t*_R_, 15 min) with 0.1% trifluoroacetic acid-MeCN (90: 10 v/v), flow rate is 6 mL/min; Carrier for each radioligand elutes through UV absorbance followed by radioactivity detectors in sequence.

4.2. HPLC analysis conditions for [^11^C]uPSEM792 radioligand.

Sunfire 5 µm C18-OBD (150 mm x 4.6 mm) eluted (*t*_R_, 9.8 min) with 0.1% trifluoroacetic acid-MeCN (90:10 v/v), flow rate is 6 mL/min; Carrier for each radioligand elutes through UV absorbance followed by radioactivity detectors in sequence.

**5. HPLC Chromatograms of Radioligand**

[A]

[B]

[C]

[D]

**Figure-S1.** HPLC Chromatograms of [^11^C]uPSEM792. (**A**) Preparative HPLC chromatogram. (**B**) Analytical HPLC chromatogram. (**C**) Analytical HPLC chromatogram for [^11^C]uPSEM792 co-injected with reference uPDSEEM792. (**D**) Analytical HPLC chromatogram for formulated [^11^C]uPSEM792 at 1 h after preparation.

**6. LC-MS/MS-MS Spectra of carrier in [^11^C]uPSEM792**


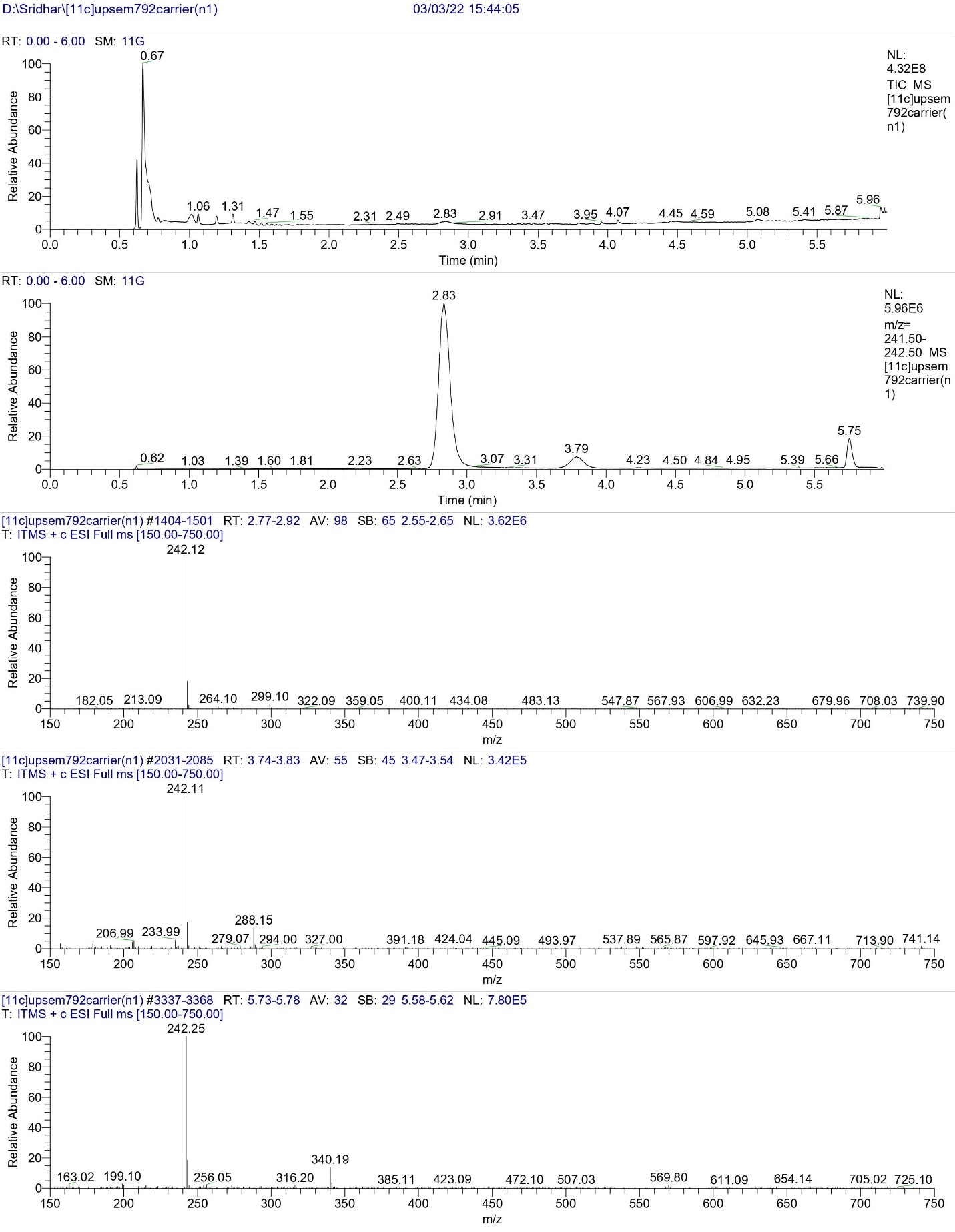


Mass of carrier-[^11^C]uPSEM792

[^11^C]uPSEM792 carrier peak


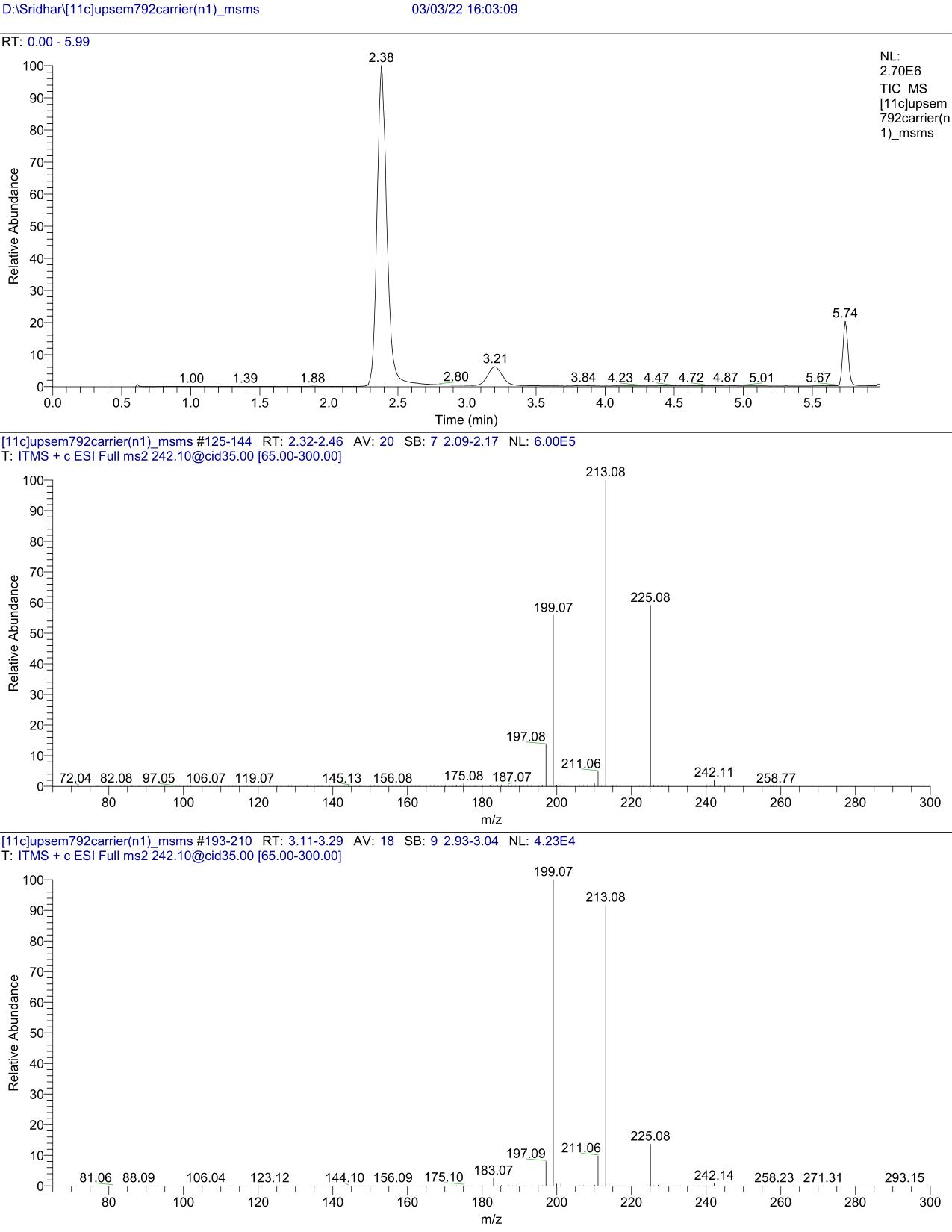


Fragmentation of carrier-[^11^C]uPSEM792

**Figure-S2.** LC-MS/MS-MS Spectra of carrier in [^11^C]uPSEM792.


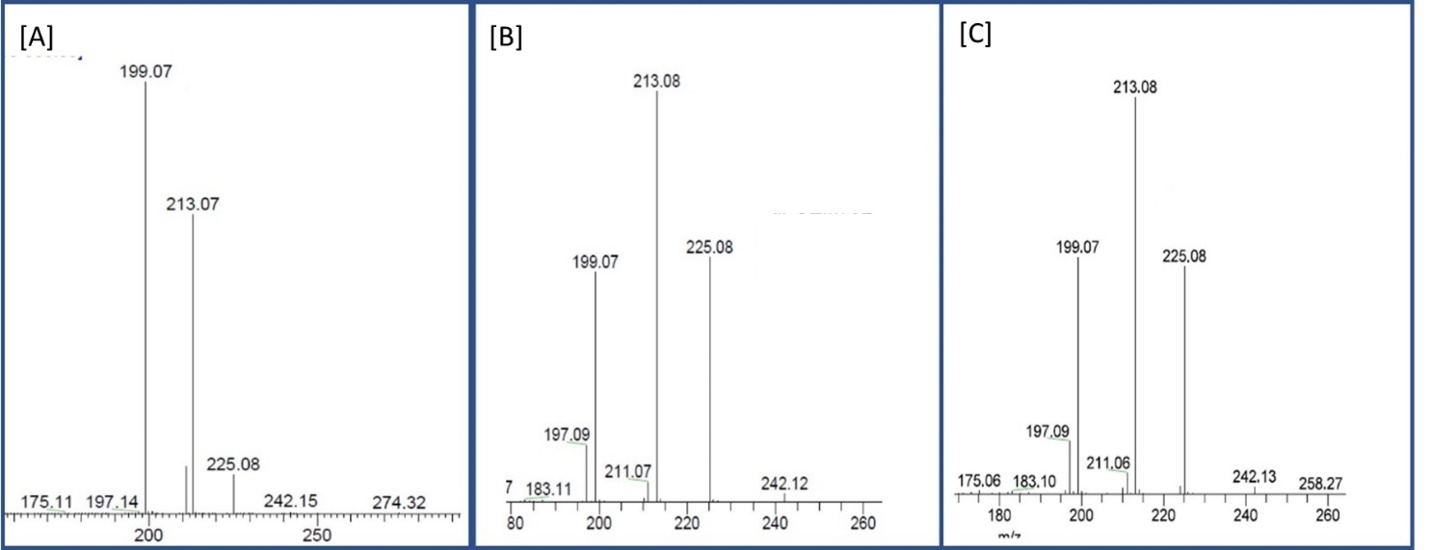


[^11^C]uPSEM792

carrier

uPSEM792

uPSEM793

**Figure-S3.** (**A**) MS-MS Spectra of PSEM793. (**B**) MS-MS Spectra of PSEM793. (**C**) MS-MS spectra of [^11^C]uPSEM792 carrier.

**7. In-vitro binding affinity studies**

The affinity of uPSEM792 for the PSAM^4^-GlyR was evaluated using of a membrane-binding assay with[3H]ASEM as the radioligand, and the detailed procedure was explained in the experimental section of the main manuscript.

**Log[drug]**

uPSEM792

**Figure S4.** Determination of the affinity (1/*K*_i_) of uPSEM792 for PSAM^4^-GlyR: displacement of [^3^H]ASEM from PSAM^4^-GlyR by uPSEM792 (error bars are SD for *n* = 3).

**8. *Ex vivo* studies**

**Table S1.** Experimental design and parameters that governed the *ex vivo* and *in vitro* study

|  |  |  | Rat-1  (Baseline) |  | Rat-2  (Baseline) |  | Rat-3  (Blocked) |  | *In vitro* |  |
| --- | --- | --- | --- | --- | --- | --- | --- | --- | --- | --- |
|  | Rat weight (g) |  | 380 |  | 370 |  | 378 |  | - |  |
|  | Injected dose (MBq) |  | 44.9 |  | 43.3 |  | 45.1 |  | - |  |
|  | Molar activity at inj. (MBq/µmol) |  | 66082 |  | 66082 |  | 66082 |  | - |  |
|  | Injected mass (nmol/kg) |  | 1.67 |  | 1.77 |  | 1.81 |  |  |  |
|  | MeCN extraction (%) |  |  |  |  |  |  |  | - |  |
|  | *Ex vivo* plasma |  | 78.8 |  | 75.8 |  | 82.7 |  | - |  |
|  | *Ex vivo* brain |  | 77.2 |  | 77.5 |  | 75.4 |  | - |  |
|  | *In vitro* fresh rat brain |  | - |  | - |  | - |  | 88 |  |
|  | Whole blood |  | - |  | - |  | - |  | 89 |  |
|  | plasma |  | - |  | - |  | - |  | 91 |  |
|  | HPLC Composition (%) |  |  |  |  |  |  |  |  |  |
|  | *Ex vivo* plasma |  | 43.0 |  | 37.9 |  | 37.1 |  | - |  |
|  | *Ex vivo* brain |  | 78.3 |  | 75.6 |  | 68.3 |  | - |  |
|  | *In vitro* fresh rat brain |  | - |  | - |  | - |  | 92 |  |
|  | Whole blood |  | - |  | - |  | - |  | 82 |  |
|  | plasma |  | - |  | - |  | - |  | 83 |  |
|  | Tissue concentration (SUV) |  |  |  |  |  |  |  |  |  |
|  | *Ex vivo* whole blood (SUV) |  | 0.141 |  | 0.163 |  | 0.163 |  | - |  |
|  | *Ex vivo* plasma parent (SUV) |  | 0.063 |  | 0.064 |  | 0.064 |  | - |  |
|  | *Ex vivo* brain (SUV) |  | 0.407 |  | 0.468 |  | 0.401 |  | - |  |
|  | *Ex vivo* brain (nM) |  | 0.68 |  | 0.83 |  | 0.72 |  | - |  |

**9. PET imaging in monkeys: experimental parameters**

**Table S2.** Experimental design and parameters that governed PET imaging in monkeys.

| [^11^C]uPSEM792 | | Baseline (BL) | Baseline (BL) |
| --- | --- | --- | --- |
|  |  | Non-transduced healthy monkey | PSAM^4^-GlyR monkey |
| Monkey | ID | H811A | DN4R |
|  | Gender | Male | Male |
|  | Weight (kg) | 9.6 | 11.40 |
| Scan Duration (min) | | 120 | 120 |
| Blood Sampling (min) | | 120 | 120 |
| Molar Activity (mCi/μmol) | | 2862 | 1122.9 |
| Injected Activity (mCi) | | 7.7 | 8.22 |
| Injected Mass | (nmol/kg) | 0.28 | 0.64 |
|  | (µg/kg) | 0.06 | 0.15 |
| *f*_P_ (%) | | 89.29 | 89.42 |

**10. PET imaging studies**

**A B**

**C D**

**Figure S5.** Plasma parent and whole blood concentration were normal and similar between non-transduced healthy and PSAM^4^ induced monkey (A & B). The concentration (SUV) over time of monkey plasma parent and radiometabolites after injection of [^11^C]uPSEM792. Plasma unchanged radioligand and radiometabolites in non-transduced healthy monkey (**C**), and PSAM^4^-GlyR transduced monkey (**B**).
